# Metabolic and Cardiorespiratory Differences in Incremental Upper and Lower-Body Exercise in Healthy Adults—A Cross-Over Trial

**DOI:** 10.3390/sports14090383

**Published:** 2026-09-01

**Authors:** Natalie Marterer, Lorenz Immler, Martin Faulhaber

**Affiliations:** Department of Sport Science, University of Innsbruck, Fürstenweg 185, 6020 Innsbruck, Austria

**Keywords:** upper-body exercise, hand crank ergometry, cycle ergometry, VO_2_^peak^, maximal fat oxidation, exercise physiology

## Abstract

Background: Upper-body exercise is increasingly applied in athletic and exercise settings; however, acute physiological and perceptual differences compared to lower-body exercise remain insufficiently characterized. This study compared maximal performance parameters during incremental upper- and lower-body exercise in healthy adults. Methods: Seventeen moderately trained participants (eight females, nine males) completed randomized incremental tests on a hand crank ergometer (HCE) and a cycle ergometer (CE). Maximal fat oxidation (MFO), intensity at maximal fat oxidation (FAT_max_), peak oxygen uptake (VO_2_^peak^), maximal heart rate (HR_peak_), peak power output (PPO), immediate post-exercise lactate (LA_post_), and differentiated ratings of perceived exertion (RPE_breathing_ and RPE_muscles_) were assessed. Results: MFO was significantly lower during HCE compared with CE (0.26 vs. 0.55 g·min^−1^, *p* < 0.001), occurring at a lower FAT_max_ (42.6% vs. 51.6% of VO_2_^peak^_,_ *p* = 0.016). Furthermore, relative VO_2_^peak^ (34.1 vs. 48.9 mL·kg^−1^·min^−1^, *p* < 0.001), HR_peak_ (178 vs. 189 beats·min^−1^, *p* < 0.001), and relative PPO (1.8 vs. 4.1 W·kg^−1^, *p* < 0.001) were substantially reduced during HCE. While RPE_breathing_ was higher during CE (19.1 vs. 17.6, *p* < 0.001), RPE_muscles_ (19.4 vs. 19.8, *p* = 0.111) and LA_post_ (9.8 vs. 8.6 mmol·L^−1^, *p* = 0.060) did not differ significantly between modalities. No significant modality-by-sex interactions were observed for any outcome. Conclusions: These findings demonstrate pronounced modality-specific differences in metabolic, cardiorespiratory, and perceptual responses. Exercise modality should therefore be carefully considered when interpreting maximal exercise tests and prescribing training or rehabilitation programs.

## 1. Introduction

Most research on maximal fat oxidation (MFO) and peak oxygen uptake (VO_2_^peak^) physiology has focused on lower-body exercise involving large muscle groups, such as running or cycling [1,2,3,4,5,6]. In comparison, research on upper-body endurance training (UBET) and the acute physiological responses to upper-body exercise, involving smaller muscle groups, remains limited and is largely confined to rehabilitation and elite para-sport settings, for example in individuals with spinal cord injury [7,8,9]. Nevertheless, evidence indicates that UBET represents an effective complementary training modality. A recent systematic review reported a mean increase in VO_2_^peak^ of approximately 16% following UBET in healthy individuals [10], underscoring its potential relevance beyond rehabilitation and para-sports, including the prevention of frailty in aging populations [11].

Upper- and lower-body exercise elicit distinct physiological responses due to differences in muscle mass, fiber composition, and cardiovascular dynamics [10]. Key outcome parameters include maximal fat oxidation and substrate utilization, maximal cardiorespiratory performance, and subjective perception of exertion.

Upper-body exercise is associated with lower maximal fat oxidation (MFO) and a lower exercise intensity at which MFO occurs compared with lower-body exercise [12,13]. In healthy male adults, MFO during incremental arm crank exercise has been shown to be substantially lower than during cycle ergometry, with MFO occurring at a lower relative intensity during upper-body exercise [12]. Knechtle et al. [14] compared arm cranking in spinal cord-injured athletes with cycling in able-bodied cyclists and found that, although both groups achieved their highest fat oxidation at the same relative intensity (75% VO_2_^peak^), absolute fat oxidation rates were lower during arm cranking. Wheelchair athletes also exhibited higher lactate concentrations at each intensity, indicating a greater reliance on carbohydrate metabolism during upper-body exercise [14]. Furthermore, when arm and leg ergometry were compared at matched power outputs, upper-body exercise elicited higher respiratory exchange ratios, indicating lower fat oxidation and greater carbohydrate utilization compared with lower-body exercise [15]. Comparisons between different lower-body exercise modalities further demonstrate differences in fat oxidation. Studies by Achten et al. and Chenevière et al. demonstrated that fat oxidation during running is approximately 25–30% higher than during cycling, while the relative intensity at which MFO occurs is comparable between modalities [16,17].

The scientific literature consistently demonstrates that VO_2_^peak^ is significantly lower during upper-body exercise compared with lower-body exercise [18,19,20,21]. For example, VO_2_^peak^ in healthy adults is consistently lower during arm crank ergometry than during cycle ergometry, typically reaching approximately 35–40 mL·kg^−1^·min^−1^ during upper-body exercise and 50–60 mL·kg^−1^·min^−1^ during lower-body exercise [18]. This difference has been observed across age groups and sexes and persists even when both exercise modalities are performed within the same individuals [19]. Similarly, in female triathletes, VO_2_^peak^ is highest during treadmill running, intermediate during cycling, and lowest during arm ergometry [20]. In addition to these modality-specific differences in VO_2_^peak^, maximal heart rate has also been reported to be lower during upper-body exercise. In healthy adults, maximal heart rate achieved during arm crank ergometry is typically 10–20 beats per minute lower than predicted by standard age-adjusted equations based on leg exercise [22]. These differences are commonly attributed to the smaller active muscle mass and reduced cardiovascular demand associated with upper-body exercise [23].

Several studies have directly compared ratings of perceived exertion (RPE) between upper- and lower-body exercise modalities [23,24,25,26]. At equivalent relative workloads, RPE has been reported to be higher during upper-body exercise than during lower-body exercise. During incremental protocols, both overall RPE, reflecting whole-body exertion, and local RPE, referring to perceived effort in the actively engaged musculature, are elevated during upper-body modalities such as arm ergometry or handcycling. In contrast, central RPE, primarily associated with cardiorespiratory strain, has been reported to be higher during lower-body exercise, including cycling [23,24]. Additionally, combined upper- and lower-body exercise elicits perceptual responses similar to upper-body exercise alone, even at higher total workloads, whereas lower-body exercise alone produces lower overall RPE at matched exercise intensities [25,26].

Therefore, the aim of the present study was to compare metabolic, cardiorespiratory, and perceptual responses during incremental upper- and lower-body exercise in healthy adults. By directly contrasting exercise involving small (arms) versus large (legs) muscle groups, this study provides evidence-based insights relevant to exercise testing and training prescription. While a previous study by Price et al. [12] investigated maximal fat oxidation during upper- and lower-body exercise, their research was limited exclusively to a male cohort. Consequently, the present study aims to replicate these findings and extend the physiological comparison to include female participants.

Given established physiological differences between females and males, including differences in muscle mass [27], sex-specific protocols were applied, and sex was included as a fixed effect in our linear mixed-effects model. Hence, the primary hypothesis to be tested was that MFO is higher for CE than HCE, even in a mixed sample of male and female participants. However, because the primary objective was the fundamental comparison between hand crank and cycle ergometry, the study was not explicitly powered to definitively detect modality-by-sex interactions. Thus, all sex-specific findings are considered strictly exploratory.

## 2. Materials and Methods

### 2.1. Study Design

The experimental protocol comprised three test days. On test day 1, anthropometric data were collected, bioelectrical impedance analysis (BIA) was performed, and participants completed familiarization sessions on the hand crank ergometer (HCE). To minimize learning effects for this novel exercise modality, participants performed the full HCE protocol during this session, exactly as it was conducted in the subsequent experimental trials. Test days 2 and 3 included incremental exercise tests performed on the HCE and cycle ergometer (CE).

**Figure 1:**
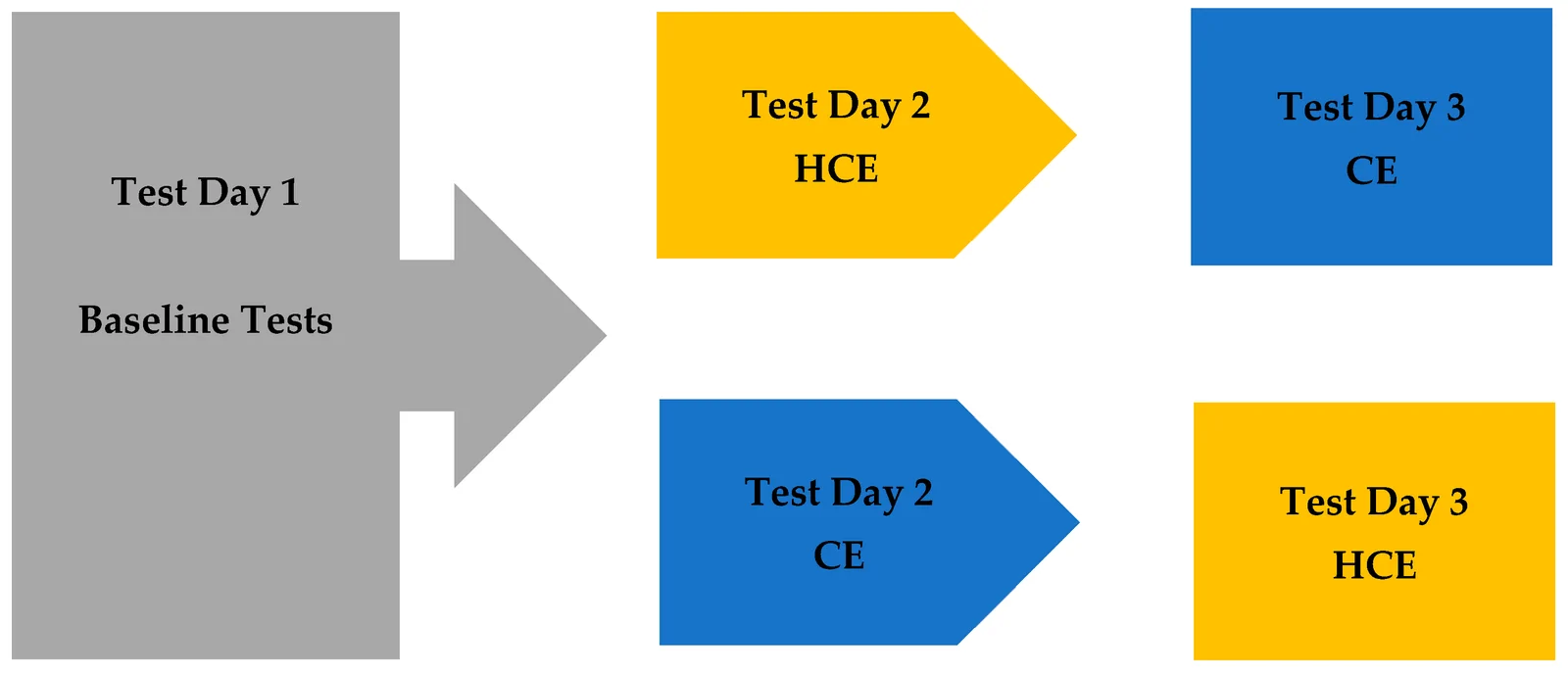
Study design. Baseline assessments are performed on Test Day 1, followed by two incremental exercise tests on separate days in randomized order using hand crank ergometry (HCE) and cycle ergometry (CE). Abbreviations: HCE = hand crank ergometry; CE = cycle ergometry.

The study followed a randomized cross-over design (Figure 1). Participants were stratified by sex, and the starting modality for the first participant of each sex was determined by drawing lots. Subsequent participants were then assigned to starting modalities in an alternating sequence to ensure balanced allocation. While the initial recruitment aimed for a perfectly balanced distribution of 20 participants (10 starting with HCE, 10 starting with CE), the final sample was reduced to 17 participants (9 males and 8 females) by dropouts during the course of the experiment. All measurements were conducted at the Department of Sport Science, University of Innsbruck (590 m above sea level). To ensure a standardized metabolic state and to control for circadian influences, participants were instructed to consume a standardized carbohydrate-rich meal on the evening prior to each test day, with the requirement to replicate this meal exactly for both testing sessions. This meal consisted of 200 g wheat noodles, 200 g tomato-based sauce, one bread roll (60 g), and one apple. All trials were performed in the morning between 7:00 a.m. and 11:00 a.m. following an approximately 12 h overnight fast. Crucially, to minimize potential diurnal variations in fat oxidation rates, each participant completed both the HCE and CE trials at the same individual clock time. The study protocol was approved by the Institutional Review Board of the University of Innsbruck (approval no. 109/22). All participants were informed about the study procedures and provided written informed consent prior to participation. The study was conducted in accordance with the Declaration of Helsinki.

### 2.2. Participants and Baseline Testing

Of the 20 recruited participants, 17 completed the study (Table 1). Three dropouts occurred due to health-related or scheduling constraints: two females (one who started with HCE and one with CE) and one male (who started with CE). This resulted in a final analysis of 17 participants (8 females, 9 males), with 9 participants having started with HCE and 8 with CE.

Participants were recruited through personal contacts. Individuals engaging in regular endurance training more than three times per week or specifically trained in upper-body exercise modalities (e.g., hand cycling, swimming, rowing, or kayaking) were excluded. Prior to inclusion, all participants completed the Physical Activity Readiness Questionnaire (PAR-Q). Additional exclusion criteria included chronic or acute musculoskeletal, cardiovascular, respiratory, neurological, or psychological diseases, smoking, pregnancy, and chronic headache or migraine. Body mass was measured before each test using a commercially available scale. Height was measured to the nearest 0.5 cm using a wall-mounted stadiometer. Body mass index (BMI) was calculated as body mass divided by height squared (kg/m^2^). Body composition was assessed during baseline testing using bioelectrical impedance analysis (BIA 101 Anniversary, Akern, Italy).

### 2.3. Laboratory Exercise Testing

Participants performed maximal incremental exercise tests on an upright cycle ergometer (CE) (Ergoline, Germany) and on a hand crank ergometer (HCE) (Ergoline, Germany) to determine MFO and VO_2_^peak^. Ergometer settings were individually adjusted for each participant. For HCE, participants were instructed to maintain shoulder contact with the seat back throughout the test to minimize trunk involvement and ensure that the workload was primarily performed by the arm musculature. During CE, standing on the pedals was not permitted. For CE testing, female participants started at 25 W with increments of 25 W every 3 min, whereas male participants started at 35 W with increments of 35 W every 3 min. For HCE testing, female participants started at 20 W with increments of 10 W every 3 min, while male participants started at 20 W with increments of 15 W every 3 min. Once the respiratory exchange ratio (RER) exceeded 1.00, stage duration was reduced from 3 min to 1 min in both exercise modalities.

A minimum recovery period of 48 h separated the two exercise tests. Gas exchange was assessed using an open-circuit spirometric system (Oxycon Pro, CareFusion, Germany), calibrated according to the manufacturer’s instructions before each test. Ventilatory parameters, including minute ventilation (VE), oxygen uptake (VO_2_), and carbon dioxide production (VCO_2_), were recorded breath-by-breath throughout testing. Heart rate (HR) was continuously monitored using a chest belt system (Polar WearLink, Finland). Ratings of perceived exertion (RPE) were obtained at the end of each stage using Borg’s 6–20 scale [28]. Prior to each test, participants were familiarized with the scale using standardized verbal anchors. Crucially, they were instructed to distinguish between two distinct sensations of strain: RPE_breathing_, representing the perceived cardiorespiratory strain (specifically focusing on shortness of breath and ventilatory effort), and RPE_muscles_, representing the perceived local physical strain, fatigue, and tension within the primary working muscle groups (arms during HCE and legs during CE). Participants provided separate ratings for each component at the end of each stage. VO_2_^peak^ was considered achieved when at least three of the following criteria were fulfilled: (I) a plateau in VO_2_ despite increasing workload (ΔVO_2_ < 2.1 mL·kg^−1^·min^−1^ between consecutive stages); (II) RER > 1.10; (III) HR exceeding 90% of age-predicted maximum; or (IV) volitional exhaustion [29]. Additionally, the test was terminated if participants were unable to maintain the required cadence (60–70 rpm for CE and 50–60 rpm for HCE) despite strong verbal encouragement. VO_2_^peak^ was defined as the highest 10 s average oxygen uptake recorded during testing. While the total test duration was not fixed, the protocol was designed to lead to volitional exhaustion within a physiologically appropriate timeframe for maximal testing, based on the individual’s performance and the predefined termination criteria. Peak power output (PPO) was calculated from the final completed workload and the fraction of time completed during the final uncompleted stage [30]. Capillary blood samples were obtained from the earlobe immediately after exercise to determine blood lactate concentration (Biosen C-Line, EKF Diagnostic, Germany).

Fat and carbohydrate oxidation rates were calculated using stoichiometric equations according to Frayn [31], assuming negligible urinary nitrogen excretion:

Fat oxidation = 1.67 × VO_2_ − 1.67 × VCO_2_ CHO oxidation = 4.55 × VCO_2_ − 3.21 × VO_2_

Gas exchange values (VO_2_ and VCO_2_) were averaged over the final 30 s of each 3 min stage to ensure metabolic stability and minimize respiratory noise. MFO was defined as the highest observed fat oxidation rate during these stages, and FAT_max_ was defined as the corresponding relative exercise intensity (%VO_2_^peak^). Stages with an RER > 1.00, as well as all subsequent stages, were excluded from the fat oxidation analysis. No additional data smoothing or curve-fitting (interpolation) was applied; MFO and FAT_max_ were determined based on the discrete values of the observed stages. Participants were instructed to refrain from vigorous physical activity for 48 h prior to testing. All tests were performed at the same time of day for each participant.

### 2.4. Statistical Analysis

Statistical analyses were performed using IBM SPSS Statistics version 28 (IBM Corp., Armonk, NY, USA). Statistical significance was set at *p* < 0.05. The principal analysis focused on MFO. FAT_max_, RER_max_, LA_post_, absolute and relative VO_2_^peak^, HR_peak_, relative PPO, RPE_muscles_, and RPE_breathing_ were analyzed as secondary exploratory outcomes. Because multiple secondary outcomes were examined without adjustment for multiplicity, their results were interpreted as exploratory.

As no a priori sample size calculation had been performed, a sensitivity analysis was conducted for MFO using G*Power version 3.1.9.7 (Heinrich Heine University Düsseldorf, Düsseldorf, Germany). Because G*Power does not directly implement the specified linear mixed-effects model, a two-tailed paired-samples t-test was used as an approximation of the within-participant comparison between exercise modalities. With a sample size of 17, an alpha level of 0.05, and a statistical power of 0.80, the minimum detectable standardized paired effect was d_z_ = 0.72.

Differences between HCE and CE were examined using linear mixed-effects models. Exercise modality, sex, test order, and the modality × sex interaction were included as fixed effects. Participant was included as a random intercept to account for repeated measurements within individuals. Models were estimated using restricted maximum likelihood. Denominator degrees of freedom were calculated using the Kenward–Roger approximation, and statistical inference was based on Type III tests of fixed effects.

For FAT_max_ and RPE_breathing_, the estimated between-participant variance of the random intercept was close to zero, and the random-intercept models did not provide an adequate solution. For these outcomes, within-participant dependency was therefore modeled using a repeated-measures structure with compound symmetry.

Estimated marginal means were calculated for HCE and CE. Modality effects are reported as adjusted mean differences between CE and HCE with corresponding 95% CIs. The modality × sex interaction was used to examine whether the effect of exercise modality differed between females and males. Main effects of sex were interpreted as overall differences between sexes across both exercise modalities and separately from the interaction effect. Test order was included to account for possible systematic effects related to whether HCE or CE was performed first.

Model assumptions were assessed by visual inspection of residual-versus-predicted-value plots and normal probability plots, supplemented by Shapiro–Wilk tests of the model residuals. Because the relative PPO model showed one comparatively large residual, a sensitivity analysis was conducted by repeating the model after excluding the participant associated with the largest absolute residual. The analysis including all participants remained the main analysis.

Local f^2^ was used as effect size because it provides a model-based estimate of the unique contribution of exercise modality within the linear mixed model, conditional on the other fixed effects and the specified covariance structure. Values of approximately 0.02, 0.15, and 0.35 were interpreted as small, medium, and large effects. Descriptive data are presented as mean ± SD. Model results are reported as estimated marginal means, adjusted mean differences, 95% CIs, F-statistics, and *p*-values.

Given that our sensitivity analysis indicated that only results with a large effect size may safely be interpreted, we inferred only those results with a local f^2^ higher than 0.35 as significant results in this study in the context of the present sample size.

## 3. Results

**Figure 2:**
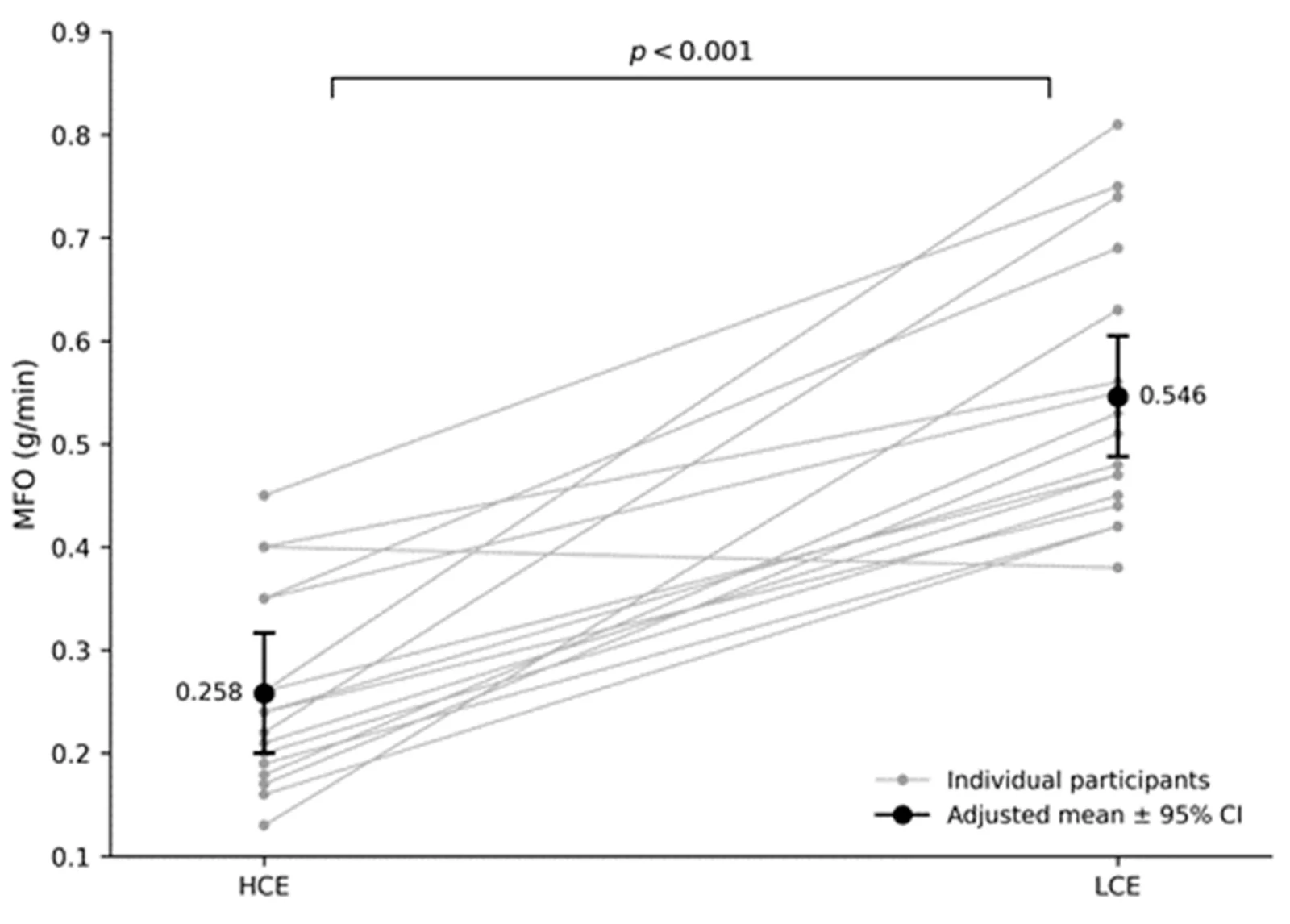
Individual maximal fat oxidation responses during hand crank and leg-cycle ergometry. Individual paired maximal fat oxidation (MFO) values during hand crank ergometry (HCE) and leg-cycle ergometry (CE) are connected by gray lines. Black circles and error bars represent model-derived estimated marginal means and corresponding 95% confidence intervals. The displayed *p*-value refers to the Type III fixed effect of exercise modality in the linear mixed-effects model.

Of 20 participants, seventeen healthy participants, including eight female and nine male participants, completed both incremental exercise tests and were included in the analyses. Model-based results are summarized in Table 2. Individual paired MFO values and the corresponding estimated marginal means are presented in Figure 2. The principal analysis focused on MFO; all remaining outcomes were analyzed as secondary exploratory outcomes. No significant test-order effect was observed for any outcome (all *p* ≥ 0.068) (Table 2).

### 3.1. Principal Analysis: Maximal Fat Oxidation

MFO was significantly lower during HCE than during CE. The estimated marginal means were 0.258 g·min^−1^ for HCE and 0.546 g·min^−1^ for CE, resulting in an adjusted mean difference of 0.288 g·min^−1^ (95% CI: 0.213–0.363; F(1,15) = 66.334, *p* < 0.001; local f^2^ = 2.29; Figure 2).

### 3.2. Secondary Exploratory Outcomes

#### 3.2.1. Metabolic Parameters

FAT_max_ was significantly lower during HCE than during CE. The estimated marginal means were 42.61% and 51.63% of VO_2_^peak^, respectively, yielding an adjusted mean difference of 9.02 percentage points (95% CI: 0.720–17.319; F(1,15) = 7.317, *p* = 0.016; local f^2^ = 0.25). A main effect of sex was observed (*p* = 0.002), whereas the modality × sex interaction was not statistically significant (*p* = 0.269).

There was no evidence of a modality effect on RER_max_. The adjusted difference between CE and HCE was −0.020 (95% CI: −0.061–0.022; F(1,15) = 0.987, *p* = 0.336; local f^2^ = 0.03). Neither the main effect of sex (*p* = 0.316) nor the modality × sex interaction (*p* = 0.559) was statistically significant.

LA_post_ was estimated to be 1.125 mmol·L^−1^ higher following CE than HCE; however, the confidence interval included zero (95% CI: −0.056–2.306; F(1,15) = 4.126, *p* = 0.060; local f^2^ = 0.14). Neither the main effect of sex (*p* = 0.276) nor the modality × sex interaction (*p* = 0.058) reached statistical significance.

#### 3.2.2. Maximal Cardiorespiratory Parameters

Absolute VO_2_^peak^ was significantly lower during HCE than during CE, with an adjusted mean difference of 987 mL·min^−1^ (95% CI: 775–1199; F(1,15) = 98.594, *p* < 0.001; local f^2^ = 3.40). A main effect of sex was observed (*p* < 0.001), whereas the modality × sex interaction was not statistically significant (*p* = 0.835).

Relative VO_2_^peak^ was also significantly lower during HCE than during CE. The adjusted mean difference was 14.888 mL·kg^−1^·min^−1^ (95% CI: 11.721–18.055; F(1,15) = 100.413, *p* < 0.001; local f^2^ = 3.46). Neither the main effect of sex (*p* = 0.094) nor the modality × sex interaction (*p* = 0.554) was statistically significant.

HR_peak_ was significantly lower during HCE than during CE, with an adjusted mean difference of 10.67 beats·min^−1^ (95% CI: 7.397–13.950; F(1,15) = 48.218, *p* < 0.001; local f^2^ = 1.66). Neither the main effect of sex (*p* = 0.752) nor the modality × sex interaction (*p* = 0.132) was statistically significant.

Relative PPO was substantially lower during HCE than during CE. The adjusted mean difference was 2.325 W·kg^−1^ (95% CI: 2.117–2.533; F(1,15) = 565.322, *p* < 0.001; local f^2^ = 19.49). A main effect of sex was observed (*p* = 0.011), whereas the modality × sex interaction was not statistically significant in the main analysis (*p* = 0.110).

The sensitivity analysis excluding the participant associated with the largest absolute residual confirmed the modality effect on relative PPO, with an adjusted mean difference of 2.387 W·kg^−1^ (95% CI: 2.222–2.552; *p* < 0.001). The modality × sex interaction became statistically significant only after this exclusion (*p* = 0.010). Because this interaction result depended on the exclusion of a single participant, it was considered unstable and was not interpreted further.

#### 3.2.3. Ratings of Perceived Exertion

RPE_muscles_ did not provide evidence of a modality effect. The adjusted mean difference was −0.361 points (95% CI: −0.816–0.093; F(1,15) = 2.868, *p* = 0.111; local f^2^ = 0.10). A main effect of sex was observed (*p* = 0.023), whereas the modality × sex interaction was not statistically significant (*p* = 0.525). RPE_muscles_ values showed pronounced clustering near the upper limit of the scale, indicating a ceiling effect.

RPE_breathing_ was significantly higher during CE than during HCE. The adjusted mean difference was 1.556 points (95% CI: 0.660–2.451; F(1,15) = 18.680, *p* < 0.001; local f^2^ = 0.64). Neither the main effect of sex (*p* = 0.298) nor the modality × sex interaction (*p* = 0.236) was statistically significant.

## 4. Discussion

The present study compared acute metabolic, cardiorespiratory and perceptual responses to incremental upper and lower-body exercise in healthy, moderately trained adults. The primary finding was that upper-body exercise on a hand crank ergometer elicited substantially lower MFO, even in a mixed sample of female and male participants, proving our primary hypothesis. We also found reduced VO_2_^peak^ (absolute and relative), lower HR_peak_, RPE_breathing_ and lower PPO compared with lower-body exercise on a cycle ergometer. Specifically, MFO during upper-body exercise was approximately 50% lower than during lower-body exercise in the total group. In addition, perceived breathing exertion was higher during lower-body exercise, whereas perceived muscular exertion did not differ between modalities. These pronounced modality-specific differences are in line with the previous literature. While these differences are likely related to smaller active muscle mass and associated cardiovascular and ventilatory demands of upper-body exercise, these potential mechanisms were not directly measured and thus remain speculative. Furthermore, given that our cohort consisted exclusively of young, healthy, and moderately trained individuals, these findings primarily have implications for exercise testing and training prescription within this specific population.

In the present study, model-derived MFO was significantly lower during HCE (EMM: 0.258 g·min^−1^) compared with CE (EMM: 0.546 g·min^−1^), resulting in an adjusted mean difference of 0.288 g·min^−1^ (95% CI: 0.213–0.363; F(1,15) = 66.334, *p* < 0.001; local f^2^ = 2.29). The overall modality effect for FAT_max_ reached formal statistical significance (*p* = 0.016); however, this secondary outcome, due to its only moderate effect size (local f^2^ = 0.25), fell below our threshold for a large effect required for robust interpretation in our sample and is not inferred to be significant in context of the current study.

Overall, these findings align with the previous literature comparing upper- and lower-body exercise. Price et al. [12] reported MFO values of 0.44 ± 0.24 g/min during arm crank ergometry and 0.77 ± 0.31 g/min during cycle ergometry, with FAT_max_ occurring at 53 ± 21% VO_2_^peak^ during upper-body exercise and at 67 ± 18% VO_2_^peak^ during lower-body exercise. Achten et al. [16] demonstrated higher MFO during treadmill running (0.65 ± 0.05 g/min) compared with cycling (0.47 ± 0.05 g/min), while FAT_max_ occurred at similar relative intensities (approximately 59–62% VO_2_^peak^).

Absolute MFO values in the present study were lower, likely reflecting differences in protocol, population, and training status; the relative pattern of reduced MFO during upper-body exercise is consistent across studies. FAT_max_ results, in contrast, are more inconsistent across the three studies (present study and [12,16]), indicating that absolute fat oxidation is strongly influenced by engaged muscle mass and exercise modality which potentially leads to a higher inter-individual variance explaining the high variability of this parameter across different studies.

Comparable modality-dependent differences have also been reported when comparing lower-body exercise modalities. A common physiological explanation for the reduced MFO during HCE is the smaller active muscle mass involved in upper-body exercise. However, because active muscle mass or limb-specific lean mass was not directly quantified in this study, this mechanism remains an inference rather than a demonstrated factor. Meanwhile, substantial inter-individual variability resulted in partially overlapping relative intensity ranges between HCE and CE in our cohort, suggesting that metabolic regulation is heavily influenced by systemic factors.

Regarding blood lactate kinetics, LA_post_ was estimated to be 1.125 mmol·L^−1^ higher following CE than HCE (9.770 vs. 8.644 mmol·L^−1^), but the confidence interval included zero (95% CI: −0.056–2.306; F(1,15) = 4.126, *p* = 0.060; local f^2^ = 0.14) and no modality × sex interaction was found (*p* = 0.058). This non-significant finding must be interpreted with caution. Given our sample size (n = 17) and the sensitivity analysis indicating that only large effects (d_z_ = 0.72) could be reliably detected, we cannot conclude a true absence of an effect or physiological equivalence. Physiologically, the estimated higher LA_post_ in CE is plausible due to the recruitment of larger muscle groups during cycling, which facilitates higher absolute workloads and a greater overall glycolytic rate, leading to increased lactate production and release into circulation [32]. Lower-body exercise (e.g., cycling, running) recruits larger muscle groups, leading to higher rates of glycolysis and lactate output, especially at maximal or near-maximal intensities [33]. The difference may not reach statistical significance due to inter-individual variability, differences in muscle fiber composition, and methodological factors such as exercise protocol and sampling timing [34].

In the total cohort, model-derived VO_2_^peak^ was significantly reduced by approximately 30% during upper-body exercise (HCE; EMM: 34.050 mL·kg^−1^·min^−1^) compared with lower-body exercise (CE; EMM: 48.938 mL·kg^−1^·min^−1^), with an adjusted mean difference of 14.888 mL·kg^−1^·min^−1^ (95% CI: 11.721–18.055; F(1,15) = 100.413, *p* < 0.001; local f^2^ = 3.46; Table 2. In line with these findings, absolute VO_2_^peak^ was also significantly lower during HCE compared with CE (EMM: 2289 vs. 3276 mL·min^−1^), representing an adjusted mean difference of 987 mL·min^−1^ (95% CI: 775–1199; F(1,15) = 98.594, *p* < 0.001; local f^2^ = 3.40; Table 2. These values are consistent with the previous literature reporting VO_2_^peak^ values of approximately 38 mL·kg^−1^·min^−1^ during arm crank exercise compared with 56 mL·kg^−1^·min^−1^ during cycling [18]. Additional studies have confirmed that VO_2_^peak^ during upper-body exercise (e.g., arm ergometry or upper-body poling) is significantly lower than during lower-body exercise (e.g., cycling or running), with typical differences ranging from approximately 25–40% [21,23]. This lower VO_2_^peak^ observed during upper-body exercise has been discussed in relation to modality-specific physiological characteristics, including a smaller active muscle mass and differences in cardiovascular responses compared with lower-body exercise. In addition, mechanical factors such as thoracic loading and differences in venous return and oxygen transport capacity have been described in the literature as factors that may be associated with lower VO_2_^peak^ values during upper-body exercise [19,21]. These physiological characteristics have been reported independent of sex; although females generally exhibit lower absolute VO_2_^peak^ values, the relative difference between upper and lower-body exercise modalities appears to be comparable between females and males [19,35]. In contrast, our descriptive data show substantial overlap in relative VO_2_^peak^ between sexes.

Regarding the chronotropic response, maximal heart rate during arm crank ergometry is typically 10–20 beats per minute lower than during leg exercise [22]. In the present study, a significantly lower HR_peak_ was also observed during HCE compared with CE (EMM: 178.265 vs. 188.939 beats·min^−1^, adjusted difference: 10.674 beats·min^−1^, 95% CI: 7.397–13.950; F(1,15) = 48.218, *p* < 0.001; local f^2^ = 1.66, Table 2). This difference occurred in parallel with the modality-specific differences observed for VO_2_^peak^ within the cardiorespiratory system, and with no evidence of a modality × sex interaction (*p* = 0.132).

Regarding maximal mechanical performance, a substantially lower PPO during HCE compared with CE was methodologically anticipated, as the upper-body workload protocol was designed with a lower starting workload and smaller wattage increments to accommodate the expected capacity limits. In line with this, relative PPO during HCE was significantly and substantially lower than during CE (EMM: 1.808 vs. 4.133 W·kg^−1^; adjusted difference: 2.325 W·kg^−1^ (95% CI: 2.117–2.533; F(1,15) = 565.322, *p* < 0.001; local f^2^ = 19.49, Table 2).

Previous studies have reported higher overall and local RPE during upper-body exercise, whereas central RPE is typically higher during lower-body exercise [23,24]. In the present study, model-derived RPE_breathing_ was significantly higher during CE compared with HCE (adjusted difference: 1.556 points, 95% CI: 0.660–2.451; F(1,15) = 18.680, *p* < 0.001; local f^2^ = 0.64, Table 2), with no modality × sex interaction (*p* = 0.236). Conversely, RPE_muscles_ did not differ significantly between modalities (EMM: 19.763 vs. 19.402, *p* = 0.111; Table 2). These findings support modality-specific perceptual responses, with greater cardiorespiratory strain during lower-body exercise. Previous studies [23,24] suggest that higher muscular strain during upper-body exercise is primarily related to a stronger contribution of local perceived exertion, which dominates overall perception of effort due to higher local metabolic stress at lower VO_2_, heart rate, and power output compared with lower-body exercise; however, this pattern was not observed in the present study. The relatively high training status of the participants, combined with the limited sample size, may have limited the ability to detect modality-specific differences in perceived muscular exertion.

## 5. Limitations

Several limitations should be acknowledged. The relatively small sample size of 17 participants and absence of an a priori power analysis limit the statistical power of the study and increase the risk of type II error for non-significant outcomes—such as LA_post_ and RPE_muscles_—and dictate that all sex-specific findings must be interpreted strictly as exploratory and descriptive. The chosen incremental test design also introduces specific physiological constraints, as physiological and metabolic responses are highly sensitive to protocol configurations. Using sex-specific absolute starting workloads and increments, rather than scaling workloads to individual fat-free mass, may have introduced additional metabolic variability. Unaccounted metabolic variance may also arise from the lack of control for the menstrual cycle phase and hormonal contraceptive use among female participants. Because fluctuating estrogen and progesterone levels are known to affect substrate metabolism and fat oxidation rates, this lack of monitoring represents a constraint for our exploratory sex-specific findings. Consequently, while attributing the significantly lower MFO and VO_2_^peak^ during HCE to a smaller active muscle mass and distinct cardiovascular limitations is highly plausible, these pathways remain speculative inferences rather than directly demonstrated mechanisms.

## 6. Conclusions

Incremental upper- and lower-body exercise elicit fundamentally different metabolic and cardiorespiratory responses in healthy adults. Upper-body exercise on a hand crank ergometer is characterized by markedly lower model-derived MFO, VO_2_^peak^, HR_peak_, and PPO compared with lower-body exercise on a cycle ergometer, highlighting the strong modality dependence of physiological load and performance outcomes. At the same time, the present results underscore the relevance of upper-body exercise testing, which remains underrepresented in both athletic and recreational training. Despite lower absolute performance values, upper-body exercise induces substantial metabolic and cardiovascular stress, supporting its role as a complementary training modality to enhance overall fitness in healthy individuals. These findings emphasize that physiological responses to upper-body exercise require modality-specific interpretation and should not be directly extrapolated from lower-body exercise data during testing or training prescription. Lastly, although our exploratory analyses did not reveal significant modality-by-sex interactions, further research with larger, adequately powered cohorts is required to fully elucidate potential sex-specific differences in upper- and lower-body metabolic and cardiorespiratory kinetics.

## Figures and Tables

**Table 1:** Demographic and anthropometric characteristics and body composition parameters of the study participants (total group and sex-specific).

	Total (*n* = 17)	Female (*n* = 8)	Male (*n* = 9)
Age (years)	26 ± 3	26 ± 3	26 ± 4
Height (cm)	174 ± 7	167 ± 3	179 ± 4
BMI	22 ± 2	22 ± 1	23 ± 3
FFM (kg)	50 ± 8	44 ± 4	56 ± 6
BCM (kg)	29 ± 6	24 ± 2	33 ± 4
MM (kg)	35 ± 7	30 ± 3	40 ± 5
FM (kg)	17 ± 4	17 ± 2	17 ± 5
VO_2_^peak^	49.2 ± 7.5	46.9 ± 5.2	51.3 ± 8.6
Data are presented as mean ± SD.			

**Table 2:** Model-based comparisons of metabolic, cardiorespiratory, and perceptual outcomes between hand crank ergometry and leg-cycle ergometry: (**A**) Estimated modality effects. (**B**) Remaining fixed effects.

(**A**)						
**Outcome**	**EMM**		**Adjusted Difference,**	**F (df_1_, df_2_)** ***p***		**Local f^2^**
	**HCE**	**CE**	**CE-HCE (95% CI)**			
MFO (g·min^−1^)	0.258	0.546	0.288 (0.213–0.363)	66.33 (1, 15)	**<0.001**	**2.29**
FAT_max_ (%VO_2_^peak^)	42.61	51.63	9.02 (0.72–17.31)	7.31 (1, 15)	0.016	0.25
RER_max_	1.09	1.07	−0.02 (−0.06–0.02)	0.98 (1, 15)	0.336	0.03
LA_post_ (mmol·L^−1^)	8.64	9.77	1.13 (−0.06–2.31)	4.12 (1, 15)	0.060	0.14
Absolute VO_2_^peak^ (mL·min^−1^)	2289	3276	987 (775–1199)	98.59 (1, 15)	**<0.001**	**3.40**
Relative VO_2_^peak^ (mL·kg^−1^·min^−1^)	34.05	48.94	14.89 (11.72–18.06)	100.41 (1, 15)	**<0.001**	**3.46**
HR_peak_ (beats·min^−1^)	178.27	188.94	10.67 (7.40–13.95)	48.22 (1, 15)	**<0.001**	**1.66**
Relative PPO (W·kg^−1^)	1.81	4.13	2.33 (2.12–2.53)	565.32 (1, 15)	**<0.001**	**19.49**
RPE_muscles_	19.76	19.40	−0.36 (−0.81–0.09)	2.87 (1, 15)	0.111	0.10
RPE_breathing_	17.56	19.11	1.56 (0.66–2.45)	18.68 (1, 15)	**<0.001**	**0.64**
(**B**)						
	**Sex,** ***p***		**Test Order,** ***p***	**Modality × Sex,** ***p***		
MFO (g·min^−1^)	0.251		0.652	0.871		
FAT_max_ (%VO_2_^peak^)	**0.002**		0.365	0.269		
RER_max_	0.316		0.675	0.559		
LA_post_ (mmol·L^−1^)	0.276		0.762	0.058		
Absolute VO_2_^peak^ (mL·min^−1^)	**<0.001**		0.617	0.835		
Relative VO_2_^peak^ (mL·kg^−1^·min^−1^)	0.094		0.068	0.554		
HR_peak_ (beats·min^−1^)	0.752		0.641	0.132		
Relative PPO (W·kg^−1^)	**0.011**		0.175	0.110		
RPE_muscles_	**0.023**		0.098	0.525		
RPE_breathing_	0.298		0.667	0.236		

*Note:* Values are estimated marginal means (EMMs). Adjusted mean differences are expressed as CE minus HCE. The F-statistics and corresponding *p*-values in Panel A refer to Type III tests of the fixed effect of exercise modality; confidence intervals refer to contrasts between estimated marginal means. Models include exercise modality, sex, test order, and the modality × sex interaction as fixed effects. Participant is included as a random intercept, except for FAT_max_ and RPE_breathing_, for which within-participant dependency is modeled using a repeated compound-symmetry covariance structure. MFO was the focus of the principal analysis. All remaining outcomes were analyzed as secondary exploratory outcomes without adjustment for multiplicity.

## Data Availability

The data presented in this study are available upon reasonable request from the corresponding author. The data are not publicly available due to privacy and ethical restrictions. During the preparation of this manuscript, the authors used ChatGPT (OpenAI, GPT-5.3) exclusively for language editing and phrasing support. OpenEvidence was used to assist in the literature search. All content was critically reviewed by the authors, who take full responsibility for the final manuscript.

## Abbreviations

The following abbreviations are used in this manuscript:

BIA	Bioelectrical impedance analysis
BCM	Body cell mass
BMI	Body mass index
CE	Cycle ergometry
CHO	Carbohydrate oxidation
CI	Confidence interval
FAT_max_	Intensity eliciting maximal fat oxidation
FFM	Fat-free mass
FM	Fat mass
HCE	Hand crank ergometry
HIIT	High-intensity interval training
HR	Heart rate
HR_peak_	Peak heart rate
IRB	Institutional Review Board
LA	Lactate
LA_post_	Immediate post-exercise lactate
MCP	Maximal cardiorespiratory performance
MFO	Maximal fat oxidation
MM	Muscle mass
PAR-Q	Physical Activity Readiness Questionnaire
PPO	Peak power output
RER	Respiratory exchange ratio
RPE	Rating of perceived exertion
RPE_breathing_	Breathing-related rating of perceived exertion
RPEmuscles	Muscular rating of perceived exertion
SCII	Spinal cord injured individuals
SD	Standard deviation
UBET	Upper-body endurance training
VCO_2_	Carbon dioxide output
VE	Minute ventilation
VO_2_	Oxygen uptake
VO_2_^peak^	Peak oxygen uptake
